# Clinical and Immunologic Manifestations of Antiphospholipid Syndrome Among Patients in King Abdulaziz Medical City (KAMC), Jeddah, Saudi Arabia

**DOI:** 10.7759/cureus.76720

**Published:** 2025-01-01

**Authors:** Rami F Magliah, Huda H Ashkar, Fayez S Alharthy

**Affiliations:** 1 Internal Medicine/Rheumatology, King Abdulaziz Medical City - Jeddah, Jeddah, SAU; 2 Internal Medicine/Rheumatology, King Saud Bin Abdulaziz University for Health Sciences College of Medicine, Jeddah, SAU; 3 Internal Medicine/Rheumatology, King Faisal Specialist Hospital and Research Centre, Jeddah, SAU

**Keywords:** anti-phospholipid syndrome, complications, immunologic, presentation, saudi arabia

## Abstract

Introduction

Antiphospholipid syndrome (APS) is a systemic autoimmune disorder marked by the presence of antiphospholipid antibodies (aPL), contributing to a heightened thrombotic risk and complications in pregnancy. This study explores the clinical and immunologic features of APS among patients at King Abdulaziz Medical City (KAMC) in Jeddah, Saudi Arabia, aiming to fill gaps in local APS data and enhance disease profiling.

Methods

A cross-sectional chart review was conducted for APS patients at KAMC from January 2008 to April 2023. Patients meeting the American College of Rheumatology (ACR) and European Alliance of Associations for Rheumatology (EULAR) APS criteria were included. Clinical manifestations, pregnancy-related complications, and immunologic profiles were documented, and gender differences were statistically analyzed.

Results

Eighty-eight patients were included, with a female predominance (76.1%). Deep vein thrombosis (35.2%) and recurrent miscarriages (33.0%) were the leading clinical manifestations in males and females, respectively. Stroke and pulmonary embolism were also prevalent, reported in 23.9% each. Anti-cardiolipin antibodies were the most common immunologic marker (56.8%), with a significant gender-based difference favoring males (76.2% vs. 50.7%). Notably, the prevalence of multiple aPL positivity was 31.8%, which was higher among males (42.9%) than females (28.4%), without statistical significance.

Conclusion

This study contributes essential epidemiological data on APS in Saudi Arabia, emphasizing gender differences and clinical heterogeneity. The findings align with other local studies, indicating younger age of onset in the Saudi population and unique gender-specific differences. High-risk APS patients, particularly males, show a higher risk of thrombosis, warranting personalized preventive strategies. These results underscore the need for tailored APS management, supporting future research to optimize APS care in this region.

## Introduction

Antiphospholipid syndrome (APS) is a rare systemic autoimmune disorder with an incidence ranging from 0.75 to 2.6 per 100,000 individuals in the general population, according to most recent international figures [1]. This condition is characterized by the presence of antiphospholipid antibodies (aPL) in the serum, leading to a prothrombotic state that significantly increases the risk of both venous and arterial thrombosis, as well as various obstetric complications. The clinical manifestations of APS can be severe, including recurrent pregnancy loss, placental insufficiency, and thrombotic events that may result in significant morbidity and mortality [2]. Another life-threatening form of APS is catastrophic APS, which is characterized by persistent antiphospholipid antibodies along with multiple vascular occlusive events [3].

The significance of APS as a systemic autoimmune disorder lies not only in its direct clinical implications but also in its potential to affect multiple organ systems. Patients with APS may experience a range of complications, including cardiovascular issues such as valvular heart disease and neurological manifestations like stroke [4]. Additionally, catastrophic APS leads to multiorgan failure [3]. Given the diverse clinical presentations and the potential for severe outcomes, APS represents a critical area of study within autoimmune diseases, necessitating ongoing research into its pathophysiology, diagnosis, and treatment strategies [5,6]. Additionally, APS is clinically divided into primary APS, which develops independently of other autoimmune conditions, and secondary APS, most frequently linked to other conditions such as systemic lupus erythematosus (SLE) [3,5].

In Saudi Arabia, the epidemiological figures of genetic disorders are significantly higher than in many other populations, which could influence the incidence and severity of autoimmune diseases, including APS. This is due to genetic predisposition of the Saudi population, characterized by a high rate of consanguinity, which may further complicate the epidemiology and clinical presentation of APS [7]. Nonetheless, local data on the clinical and laboratory features of these patients remain scarce [8], underscoring the need for further studies to better understand the unique clinical and biological features of APS in this population.

The aim of this study is to evaluate the clinical and immunologic manifestations of APS among patients attending a referral center in Jeddah, Saudi Arabia. Such data is critical to improve diagnostic accuracy and tailor treatment protocols, thereby addressing gaps in the effective management of APS within the Saudi healthcare system.

## Materials and methods

Design and settings

This cross-sectional chart review was conducted at the Department of Rheumatology of King Abdulaziz Medical City (KAMC) in Jeddah, Saudi Arabia. Medical records registered between January 1, 2008, and April 30, 2023, were extracted from the medical records department using the code for unspecified hematological disorders. The study received ethical approval from the institutional review board of King Abdullah International Research Center (approval number: IRBC/685/16 dated February 17, 2022).

Participants

We included all patients who were diagnosed or followed up at the participating center, and who fulfilled the 2023 classification criteria of the American College of Rheumatology (ACR) and the European Alliance of Associations for Rheumatology (EULAR) for the diagnosis of APS. The criteria required meeting at least one clinical criterion and one laboratory criterion, across all age groups.

Convenience sampling was utilized to select patient records. Eligible records were reviewed to extract relevant clinical and laboratory data for APS diagnosis and classification into primary and secondary subtypes.

Data collection

The primary outcome was to determine the distribution of primary and secondary APS and describe the initial presenting symptoms among the cohort. Hence, the following data were extracted: (i) Demographics: Gender and age at disease onset (defined as the age at the time of the initial symptom attributable to APS); (ii) Clinical manifestations: Deep vein thrombosis (DVT), recurrent miscarriages, cerebrovascular thrombosis (CVT), stroke, large arterial thrombosis (LAT), and upper extremity arterial thrombosis (UAT). Manifestations were confirmed through laboratory, radiological, or histopathological evidence. Doppler ultrasonography was used to establish the diagnosis of DVT, computed tomography (CT) or magnetic resonance imaging (MRI) was used for the diagnosis of CVT, and stroke, and arteriography was used to confirm a diagnosis of LAT and UAT. Manifestations established based on a clinical basis only were excluded; (iii) Pregnancy-related complications: Repeated miscarriages, premature birth, stillbirth, and pre-eclampsia were considered only if the patient met the preliminary diagnostic criteria for APS; and (iv) Immunologic findings: The presence of anticardiolipin (ACL) antibodies and anti-β2 glycoprotein I (anti-β2-GPI) antibodies were evaluated using standard enzyme-linked immunosorbent assay (ELISA) tests. Test results were considered positive if the titers were moderately high or above and confirmed at least 12 weeks apart, in accordance with the 2023 classification criteria for the diagnosis of APS. Lupus anticoagulant (LAC) was assessed through coagulation assays, with positivity established based on prolonged phospholipid-dependent clotting times and subsequent confirmatory tests.

Statistical methods

Metric variables were summarized as mean and standard deviation (SD), while categorical variables were presented as frequencies and percentages (%). Gender differences in clinical and laboratory characteristics were analyzed using Fisher’s exact test for categorical variables and the independent sample t-test for continuous variables. Statistical significance was set at p < 0.05. All statistical analyses were conducted using IBM SPSS Statistics for Windows, Version 21.0 (Released 2012; IBM Corp., Armonk, New York, United States).

## Results

Baseline demographics

Eighty-eight patients met the APS criteria during the study period. Of these, 21 (23.9%) were male and 67 (76.1%) were female. The mean age of the patients was 42.5 years (SD = 12), and the mean age at onset was 34.7 years (SD = 9.67), with no significant difference between genders (p > 0.80). Among the participants, 53.4% had primary APS, and 37.5% had secondary APS, with no gender-based differences (p=0.962) (Table 1).

**Table 1:** General characteristics according to gender. ^§^ P-value has been calculated using Chi-square test;  ^‡^ P-value has been calculated using independent sample t-test; ^*^ Significant at p<0.05 level.

Study variables	Overall (N=88)	Male (n=21)	Female (n=67)	t-test	P-value ^‡^
Age in years, mean ± SD	42.5 ± 12.0	42.4 ± 13.7	42.5 ± 11.6	-0.016	0.987 ^‡^
Age at onset in years, mean ± SD	34.7 ± 9.67	34.2 ± 10.1	34.9 ± 10.1	-0.252	0.802 ^‡^
APS type	n (%)	n (%)	n (%)	X2	P-value ^§^
Primary	47 (53.4%)	13 (61.9%)	34 (50.7%)	0.800	0.456
Secondary (SLE)	33 (37.5%)	06 (28.6%)	27 (40.3%)	0.938	0.441

Clinical manifestations

The most frequent clinical manifestations, by domain, included macrovascular venous disorders, observed in 47 (53.4%) patients, and were more frequent among male participants (71.4%) than female (47.8%); however, the difference did not reach statistical significance (p=0.080). Additionally, macrovascular arterial manifestations involved 25 (28.4%) patients, being more frequent among male patients (38.1%) than females (25.4%) with no statistically significant difference. Obstetric complications were also common, reported in 30 (44.8%) female patients (Table 2).

**Table 2:** Clinical domains in relation to gender. ^§^ P-value has been calculated using Fischer's Exact test; ^** ^Significant at p<0.05 level

Variables	Overall (N=88), n (%)	Male (n=21), n (%)	Female (n=67), n (%)	p-value ^§^
Macrovascular venous	47 (53.4%)	15 (71.4%)	32 (47.8%)	0.080
Obstetric complications	30 (34.1%)	N/A	30 (44.8%)	N/A
Macrovascular arterial	25 (28.4%)	08 (38.1%)	17 (25.4%)	0.259
Hematology	13 (14.8%)	01 (04.8%)	12 (17.9%)	0.177
Cardiac valve	02 (02.3%)	0	02 (03.0%)	1.000
Microvascular	01 (01.1%)	01 (04.8%)	0	0.239

Detailing the clinical manifestation (Table 3), the most frequent among all patients were DVT (35.2%), repeated miscarriages (33.0%), pulmonary embolism (23.9%), and stroke (23.9%). In female patients, repeated miscarriages were the predominant manifestation, observed in 43.3% of cases, while DVT was the most common manifestation among males, present in 42.9%. Male patients also showed a significantly higher frequency of lower arterial thrombosis compared to females (19.0% vs. 3.0%; p = 0.027). Other frequent manifestations, such as stroke, pulmonary embolism (PE), and arthralgia, were similarly distributed between genders (p > 0.05 for all).

**Table 3:** Frequency of clinical manifestation in relation to gender. ^§^ P-value has been calculated using Fischer's Exact test; ^**^ Significant at p<0.05 level. AVN: avascular necrosis; DVT: deep venous thrombosis; MI: myocardial infarction; PHTN: pulmonary hypertension; PE: pulmonary embolism

Variables	Overall (N=88), n (%)	Male (n=21), n (%)	Female (n=67), n (%)	P-value ^§^
DVT	31 (35.2%)	09 (42.9%)	22 (32.8%)	0.440
Repeated miscarriage	29 (33.0%)	N/A	29 (43.3%)	N/A
PE	21 (23.9%)	05 (23.8%)	16 (23.9%)	1.000
Stroke	21 (23.9%)	04 (19.0%)	17 (25.4%)	0.770
Arthralgia	17 (19.3%)	04 (19.0%)	13 (19.4%)	1.000
Thrombocytopenia	10 (11.4%)	01 (04.8%)	09 (13.4%)	0.440
Epilepsy	09 (10.2%)	01 (04.8%)	08 (11.9%)	0.680
Arthritis	09 (10.2%)	02 (09.5%)	07 (10.4%)	1.000
Lower Arterial Thrombosis	06 (06.8%)	04 (19.0%)	02 (03.0%)	0.027 **
Splanchnic Thrombosis	05 (05.7%)	02 (09.5%)	03 (04.5%)	0.589
PHTN	05 (05.7%)	0	05 (07.5%)	0.332
Preeclampsia	01 (01.1%)	N/A	01 (01.5%)	N/A
Valvular Heart Disease	04 (04.5%)	0	04 (06.0%)	0.569
Cerebral Vein Thrombosis	04 (04.5%)	01 (04.8%)	03 (04.5%)	1.000
Migraine	04 (04.5%)	0	04 (06.0%)	0.569
Stillbirth	04 (04.5%)	N/A	04 (06.0%)	N/A
Upper Arterial Thrombosis	03 (03.4%)	0	03 (04.5%)	1.000
Mesenteric Ischemia	03 (03.4%)	02 (09.5%)	01 (01.5%)	0.140
Renal infarction	03 (03.4%)	02 (09.5%)	01 (01.5%)	0.140
Hemolytic Anemia	03 (03.4%)	0	03 (04.5%)	1.000
MI	02 (02.3%)	01 (04.8%)	01 (01.5%)	0.422
Coronary Artery Disease	02 (02.3%)	0	02 (03.0%)	1.000
AVN	02 (02.3%)	0	02 (03.0%)	1.000
Livedo Reticularis	02 (02.3%)	02 (09.5%)	0	0.055
Superficial Thrombophlebitis	02 (02.3%)	01 (04.8%)	01 (01.5%)	0.422
Systemic Vasculitis	01 (01.1%)	01 (04.8%)	0	0.239
Dilated Cardiomyopathy	01 (01.1%)	0	01 (01.5%)	1.000

Immunological profiles

The most frequent immunological manifestation (Table 4) in the overall cohort was the presence of anti-cardiolipin antibodies (56.8%), followed by lupus anticoagulant (45.5%) and anti-β2 glycoprotein I antibodies (23.9%). Among male patients, anti-cardiolipin antibodies were significantly more prevalent compared to female patients (76.2% vs. 50.7%; p = 0.047). The presence of lupus anticoagulant was similar between genders (38.1% in male patients vs. 47.8% in female; p = 0.438). Additionally, a higher proportion of male patients had more than one positive immunological marker compared to female patients; however, this difference did not reach statistical significance (42.9% vs. 28.4%; p = 0.283).

**Table 4:** Laboratory results according to gender. ^§^ P-value has been calculated using Fischer's Exact test; ^**^ Significant at p<0.05 level

Variables	Overall (N=88), n (%)	Male (n=21), n (%)	Female (n=67), n (%)	P-value ^§^
Lupus anticoagulant	40 (45.5%)	08 (38.1%)	32 (47.8%)	0.438
Anti-cardiolipin	50 (56.8%)	16 (76.2%)	34 (50.7%)	0.047 **
Anti-B2-Glycoprotein 1 antibodies	21 (23.9%)	05 (23.8%)	16 (23.9%)	1.000
More than one positive	28 (31.8%)	09 (42.9%)	19 (28.4%)	0.283

## Discussion

Clinical and laboratory data on APS patients in Saudi Arabia are limited. Accumulating local data is crucial to form a more accurate picture of the disease in this population, leading to improved diagnostic accuracy and the development of tailored treatment protocols. This, in turn, ultimately addresses gaps in its effective management within the Saudi healthcare system.

This study explored the clinical and immunological manifestations of APS among patients at KAMC in Jeddah. The cohort was marked by an obvious female predominance. The most prevalent clinical manifestation was DVT, affecting 35.2% of the cohort, with a higher incidence in male patients compared to patients, though the difference was not statistically significant. Recurrent miscarriage was the most common manifestation among female patients, observed in 43.3% of cases. Stroke was another significant complication, affecting 23.9% of patients, with no noticeable gender differences, although lower arterial thrombosis was significantly more frequent in male patients (19.0%) compared to female patients (3.0%). PE also emerged as a frequent complication, present in 23.9% of patients with similar distribution across genders. In terms of immunologic profiles, anti-cardiolipin antibodies were the most frequent marker, detected in 56.8% of patients, with a significantly higher male prevalence (76.2%) than female (50.7%). Additionally, more male patients exhibited multiple positive immunologic markers, although this was not statistically significant. These findings emphasize the diverse and gender-specific clinical presentations of APS, underscoring the importance of tailored management strategies for different patient subgroups.

Demographic features of APS patients

We observed a clear female predominance among our cohort with a relatively young age of onset at approximately 35 years. Other local data show an even younger age of onset. Algahtani et al. conducted a similar study in Riyadh, reporting the clinical and laboratory profiles of APS in a cohort of 80 patients, among whom 22 (27.5%) met the Sapporo criteria, which had significant implications for the diagnosis and epidemiology of the disease [8]. Their results showed a relatively younger age at diagnosis (mean age = 28.5 years) compared with our cohort. Additionally, the proportion of female participants was relatively higher (90%) in Algahtani et al.’s cohort compared to that of the current study (76%) [8]. Gender distribution of APS in international data shows a higher frequency in women, with female-to-male ratios ranging from 2:1 to 10:1 in studies including SLE and obstetric APS, but this ratio approaches 1:1 when these factors are excluded. In pediatric APS, the female-to-male ratio is nearly equal, and among the elderly, males outnumber females [1].

Regarding age at onset, comparison with international data suggests an earlier onset among the Saudi population (including our series and that by Algahtani et al. [8]). Indeed, international literature shows that APS incidence tends to peak later in life with a mean age of diagnosis around 50 years, despite varying incidence across the populations [1]. On the other hand, while we observed no difference in the age of onset between genders, data from the literature shows an earlier onset in women, with incidence peaking between ages 30-39 years and 70-79 years, whereas in men, incidence peaks at 55-64 years and 70-79 years [1]. An earlier onset of APS in the Saudi population implies a prolonged risk period for complications, necessitating sustained healthcare support and highlighting the need for research into population-specific factors.

Etiology of APS in the study cohort

We observed that primary APS constituted most of the cohort. Etiologically, APS is classified into primary and secondary forms. Primary APS occurs independently, without any associated autoimmune disorder, while secondary APS is associated with other conditions, most commonly SLE [5,9]. Epidemiologically, primary APS has an estimated prevalence of 40-50 cases per 100,000 individuals, whereas secondary APS, particularly in SLE patients, shows a prevalence of 30-50% for antiphospholipid antibodies [3,10,11]. This is in agreement with our results showing that one-third of the patients had a secondary form, with SLE being the unique etiology observed in our cohort. SLE is one of the most frequent etiologies of secondary APS. Besides SLE, other frequent etiologies include other autoimmune diseases such as rheumatoid arthritis and Sjögren's syndrome, which contribute to a hypercoagulable state due to the presence of antiphospholipid antibodies [9,12]. Other potential triggers for secondary APS can include infections, malignancies, and certain medications [13-15]. The clinical manifestations of both forms include recurrent thrombosis and pregnancy complications, but secondary APS is often associated with more severe clinical outcomes due to the underlying disease [14,15].

Therefore, determining the etiology of APS and the eventual triggers has significant implications for the patient as it influences both the management strategy and the prognosis. The EULAR recommendations for the management of APS highlight that management strategies for primary APS focus primarily on preventing thrombosis, often with long-term anticoagulation, depending on the risk profile (e.g., high-risk antibody profiles). In contrast, the treatment for secondary APS, especially when associated with SLE, involves managing both the autoimmune disorder (e.g., using hydroxychloroquine or immunosuppressants) and the prothrombotic state [14]. Recent studies further confirm the difference in clinical approaches, showing that secondary APS, particularly when associated with SLE, tends to present with more severe systemic manifestations, requiring more aggressive immunomodulatory treatments alongside standard anticoagulation. This is due to the compounded risk from both APS and the underlying autoimmune disorder [16].

Thrombotic complications of APS

This study found a high prevalence of thrombotic manifestations, with DVT being the most frequent (35.2%) and more common in male patients (42.9%) than female patients (32.8%), although the difference was not statistically significant. The male-specific risk of thrombotic complications probably corresponds to a specific phenotype of APS named male thrombotic phenotype with a higher risk of venous thromboembolic events [16]. This emphasizes the importance of early thrombotic risk assessment, particularly in male patients with APS.

Thrombotic events including DVT are consistently reported as the most frequent clinical manifestation of APS. In the series by Algahtani et al., both Sapporo-confirmed and clinically diagnosed subgroups showed a higher prevalence of vascular thrombosis and deep vein thrombosis than in our cohort [8]. In another cohort by Duarte-García et al., from the United States, 100% of the patients had thrombosis and DVT was observed in 48% of the patients [10]. A 14-year nationwide epidemiological study from Taiwan, involving 19,163 newly diagnosed participants, reported a prevalence of DVT at 6.1%, significantly higher than in control individuals without APS (0.3%) [17]. Another prospective study from the United States showed DVT to be the most frequent clinical manifestation among incident APS cases, with a slight difference between cases meeting Sydney criteria (42%) and the total cohort (48%) [10]. Several factors could explain the relative variability in thrombotic manifestations across studies, including differences in disease severity, comorbidities such as SLE, and variations in treatment protocols like anticoagulation use. Geographic and ethnic factors may also contribute, as well as differences in study methodologies, such as diagnostic criteria and cohort selection. These disparities emphasize the need for tailored APS management to account for both regional and patient-specific risk factors.

The occurrence of thrombotic events in APS probably reflects a higher disease activity. Evidence shows that patients with thrombotic primary APS exhibit significantly elevated levels of both inflammatory and coagulation markers, suggesting that thrombotic primary APS is associated not only with hypercoagulability but also with a persistent inflammatory state, particularly in patients with venous thrombosis [18]. This association is also relevant in the other direction, where VTE can be the presenting symptom for APS. This is mostly evident among young patients presenting with a first episode of idiopathic VTE [19]. This highlights the role of both screening and preventative strategies, notably the importance of optimal therapeutic anticoagulation in managing these patients to prevent further complications.

Obstetrical complications

Among the female patients, recurrent miscarriages were the most common clinical manifestation (43.3%), representing the major obstetric complication of APS, while preeclampsia was reported in one patient (1.5%). This finding underscores the significance of APS as a leading cause of pregnancy complications. In agreement with our findings, the series by Algahtani et al. also reported a high prevalence of obstetrical complications (51.7% in clinically diagnosed group), and first-trimester abortion was the most frequent complication reported in 18.2% and 31.0 in Sapporo confirmed and clinically diagnosed groups respectively [8]. In the population-based cohort of incident cases by Duarte-García et al., the prevalence of pregnancy morbidity was lower (17%); however, this cohort was characterized by a lower female ratio (55%) and an older population (mean age = 54 years) [10]. A study by Ali and Omar, involving women in the Makkah region, Saudi Arabia, found that antiphospholipid antibodies increased with the number of miscarriages and gestational age at the time of miscarriage, remaining high in the first four years post-miscarriage before declining [20].

Obstetric APS constitutes a major clinical entity of APS and is a leading cause of repeated pregnancy loss and late pregnancy complications linked to placental damage. It is defined by a set of clinical and laboratory criteria; however, the nomenclature of obstetric APS applied specifically for cases meeting the Sydney criteria for obstetric complications, without prior thrombosis. Cases with incomplete clinical or laboratory criteria are classified either as obstetric morbidity APS (OMAPS) or non-criteria OAPS (NC-OAPS) [21]. The pathogenesis of APS in pregnancy is characterized by a complex interplay of immune dysregulation, endothelial dysfunction, and placental pathology. The presence of antiphospholipid antibodies significantly impacts pregnancy outcomes, leading to complications such as recurrent pregnancy loss, preeclampsia, and thromboembolic events [21,22]. Understanding these mechanisms, including emerging factors like extracellular vesicles and micro-RNAs, is crucial for the effective management of pregnant women with APS, ultimately aiming to improve maternal and fetal health outcomes [22]. Future research should continue to explore these underlying mechanisms to develop targeted therapies and interventions.

The management of obstetric APS involves preconception counseling to assess prior pregnancy complications, thrombosis, and other risk factors. For high-risk aPL profiles, low-dose aspirin (LDA) alone is recommended, while recurrent miscarriage cases often require LDA plus heparin at prophylactic doses. Refractory cases may benefit from added treatments like hydroxychloroquine (HCQ), low-dose steroids, or intravenous immunoglobulin. After delivery, heparin is continued for six weeks postpartum to prevent maternal thrombosis, with adjustments based on individual risk profiles and previous pregnancy outcomes [14,21].

Stroke and arterial thrombosis

Stroke was reported in 23.9% of the cohort, with no significant gender difference, while lower arterial thrombosis was significantly more frequent in male patients (19.0%) than female patients (3.0%). Stroke and transient ischemic accidents are reportedly the most frequent arterial events in APS [23]. This is consistent with a cohort study involving 361 patients, among whom 25.8% developed stroke during a three-year follow-up. The majority of these cases were ischemic stroke (23.5%), followed by transient ischemic attack (TIA) (1.7%) and cerebral venous sinus thrombosis (1.1%) [24]. Population-based data show that the prevalence of stroke is reported to be 56-fold higher in APS patients than in individuals without APS [17]. A meta-analysis involving 17 cohort studies showed that APS is associated with a -1.76 hazard ratio of stroke (95%CI: 1.39-2.21), which increases to 14.70 (7.56-28.56) in case of associated autoimmune diseases such as SLE [25]. The occurrence of stroke as a complication of APS complicates treatment, given the likelihood of higher morbidity and recurrent thrombotic events in these patients, besides the limited evidence on optimal antithrombotic therapy for secondary prevention [26], highlighting the importance of primary prevention among already diagnosed APS cases.

Immunological profiles

In the present cohort, the most frequent immunologic marker was anti-cardiolipin antibodies, present in 56.8% of the overall cohort, followed by lupus anticoagulant (45.5%) and anti-β2-glycoprotein I antibodies (23.9%). Additionally, approximately one-third of patients had more than one positive immunologic marker, suggesting a high prevalence of multiple antibody positivity in this population. In Algahtani et al.'s series, significant differences were observed in the immunological profiles between the Sapporo-confirmed and clinically diagnosed cases of APS; lupus anticoagulant was positive in only one case, representing 1.3% of the total cohort. In contrast, repeat-positive anticardiolipin antibodies were found in 31.3% of all patients, with a much higher prevalence in Sapporo-confirmed cases (95.5%) compared to clinically diagnosed cases (6.9%) [8]. This is still lower than our cohort showing higher rates of positive immunologic profiles. The literature reveals significant heterogeneity in the serological profiles of APS patients, which determines the risk stratification and conditions of the resulting therapeutic approach [14]. A population-based study from a county in Minnesota, United States, found proportions of positive lupus anticoagulant (75%), anticardiolipin (70%), and anti-β2GPI antibodies (56%) among incident cases meeting Sydney criteria [19]. A study comparing a cohort of 44 elderly patients with APS with the Euro-Phospholipid cohort (n=1000) showed higher positivity rates in the elderly cohort for anticardiolipin antibodies (73% versus 88% in the Euro-Phospholipid cohort) and lupus anticoagulant (70% versus 54%), and 43.2% of the cohort were triple positive antiphospholipid antibodies [27].

Although antiphospholipid antibodies are central to APS pathogenesis, they are not consistently present in patients' sera, leading to a diagnosis sometimes based on clinical presentation alone. A prospective French cohort of 87 patients found that 17 (19.5%) were seronegative but diagnosed based on strong clinical suspicion, while 11 were asymptomatic despite positive antiphospholipid antibodies (aPL). The study also identified a correlation between non-conventional antibody positivity, particularly anti-phosphatidylserine/prothrombin (anti-PS/PT) antibodies, APS severity, and lupus anticoagulant positivity [28]. Another multicenter, long-term follow-up study found seronegativation in about 9% of initially positive patients, particularly among those with single aPL positivity [29]. These data demonstrate the heterogeneity in the immunological profiles of APS patients, which compounds the diagnosis and management. Furthermore, the prevalence of antiphospholipid antibodies in the general population remains unclear. Up to 10% of healthy donors test positive, though fewer than 1% remain positive after a year. High-risk profiles are rare in healthy people but persist in 20-30% of SLE patients. Prevalence varies among nonautoimmune groups, particularly those with pregnancy complications, thrombosis, myocardial infarction, or stroke [23]. Taken together, these findings underscore the importance of repeated and comprehensive serologic evaluations, alongside clinical judgment, to ensure accurate diagnoses and personalized therapeutic strategies for APS.

Interestingly, a recent multicenter French cohort study of 253 patients identified four distinct phenotypes based on the clinical and immunological profiles [16]. These include asymptomatic antiphospholipid antibody carriers with low event risk, male thrombotic phenotype with higher risk of venous thromboembolic events, female obstetrical phenotype experiencing both obstetric and thrombotic events, and high-risk APS group characterized by younger patients, triple antibody positivity, and a higher frequency of arterial events. This indicates that immunological profiles are intricately linked to clinical phenotype and disease activity, influencing prognosis and management strategies.

In seronegative APS (SN-APS) patients, despite the absence of traditional markers like anti-cardiolipin, anti-β2-GPI, and lupus anticoagulant, new proteomic approaches have enabled the detection of alternative antibodies, such as anti-vimentin/cardiolipin, using methods like ELISA and TLC immunostaining [30]. These techniques underscore the heterogeneity of SN-APS and reveal its complex antibody profile while forecasting novel discoveries of other non-conventional autoantibodies that may further refine diagnosis and improve patient stratification in the future.

Limitations and strengths

This study has several limitations, including its small cohort size, lack of treatment and outcome data, absence of comparative analysis between primary and secondary APS, and limited clinical characterization of the cases (e.g. high, medium, low risk; catastrophic APS). Despite these limitations, the cohort provides valuable clinical and immunological insights, contributing to local and regional epidemiological data on APS. This study enhances the understanding of APS profiles in the country, supporting future research and better patient management tailored to regional characteristics. Nonetheless, the study addresses a critical gap in the local data on APS aimed at improving disease profiling notably clinical manifestations and immunologic characteristics.

## Conclusions

Our cohort showed a relatively younger onset age compared to international data, in line with other regional studies, suggesting possible genetic or environmental influences that merit further exploration. Secondary APS was observed in about one-third of cases, exclusively linked to SLE. Major clinical features included deep vein thrombosis in male participants and recurrent miscarriage in female participants, alongside high rates of stroke and pulmonary embolism, underscoring APS’s significant disease burden. Elevated anti-cardiolipin antibody positivity, particularly in male patients, indicates the eventual need for targeted screening strategies, especially given the younger onset. Optimizing primary prevention of thromboembolic events could reduce morbidity, and additional genetic research may reveal mutations relevant in this population with high consanguinity. Further local multicenter studies are warranted to confirm these conclusions and to develop actionable strategies and specific recommendations for clinical practice.
